# Implementing targeted vaccination activities to address inequalities in vaccination: a qualitative study

**DOI:** 10.1093/pubmed/fdaf006

**Published:** 2025-01-27

**Authors:** Fay Bradley, Pauline A Nelson, Jo Dumville

**Affiliations:** Division of Nursing, Midwifery and Social Work, School of Health Sciences, The University of Manchester, Jean McFarlane Building, Oxford Road, Manchester M13 9PL, UK; Sheffield University Management School, The University of Sheffield, Conduit Road, Sheffield, S10 1FL, UK; Division of Nursing, Midwifery and Social Work, School of Health Sciences, The University of Manchester, Jean McFarlane Building, Oxford Road, Manchester M13 9PL, UK

**Keywords:** COVID-19, public health, vaccination

## Abstract

**Background:**

As the UK COVID-19 vaccination programme progressed, greater emphasis was placed on the implementation of localized targeted vaccination activities to address inequalities in vaccination coverage. This study examines one UK region’s approach to the delivery of targeted vaccination activities and identifies key factors influencing implementation.

**Methods:**

Qualitative interviews were conducted with a purposive sample of key individuals involved in vaccination delivery across Greater Manchester (GM). A rapid analysis approach was adopted. A template based on the Consolidated Framework for Implementation Research helped to guide analysis.

**Results:**

Twenty-seven participants were interviewed, from nine of the 10 GM localities. All areas planned to implement targeted vaccination activity, but size and scope varied. Five factors influencing the implementation of targeted vaccination approaches were identified: (i) integrated working; (ii) data availability and monitoring, (iii) engagement with populations and communities, (iv) resources and infrastructure, and (v) external policies and procedures.

**Conclusion:**

The study provides wider lessons for future public health interventions around the need for collaborative working, adequately resourced community engagement, appropriate data, long-term workforce/system planning and coherence in policy and messaging. These findings have led to the generation of six key insights for the implementation of future targeted vaccination programmes.

## Introduction

In December 2020, the UK became the first country to administer an approved COVID-19 vaccination.^1^ Guided by the Joint Committee for Vaccination and Immunization, the initial phase of the UK COVID-19 vaccination programme followed a prioritization list based on age; care home residents and staff, and frontline health and social care workers were also prioritized, as were younger adults with underlying health conditions.^2^ The early focus of the programme was on delivery at pace and scale. Various delivery models were adopted including hospital hubs, mass vaccination centres, general practitioner (GP) and primary care network (PCN)-led delivery sites as well as community pharmacies.^3^ However, the emergence of COVID-19 vaccination data enabled the identification of communities, populations, and areas of low coverage. The Office for National Statistics reported in May 2021 that disparity in COVID-19 vaccination rates was associated with several factors, including socioeconomic deprivation, ethnicity, age, and religion.^4^ These data, along with a corresponding shift from the initial centralized ‘top-down’ vaccination approach to a more localized focus, led to the development of tailored, targeted delivery approaches involving collaboration between Local Authorities and National Health Service (NHS) providers.^5^

Here we define targeted vaccination activities as those separate from mass vaccination offers that aimed to increase vaccination rates in specific groups or communities. Activities can include information provision, engagement and communication and/or use of alternative neighbourhood-based locations for vaccination delivery (e.g., through home visits, pop-up or mobile clinics).^6^

The Greater Manchester (GM) region, in the Northwest of England, has a spectrum of deprivation and is ethnically diverse. The region saw high rates of COVID-19 infection, and disproportionally high virus-related morbidity and mortality, with COVID-19 mortality rates 25% higher than in England as a whole.^7^ Previous research has demonstrated ethnic inequalities in the receipt of a COVID-19 vaccination in GM, with lower levels of vaccination in 15 of 16 minority ethnic groups, when compared with the ‘White British’ ethnic group; these inequalities were also found to exceed inequalities in flu vaccination.^8^

Although there is evidence for the implementation of targeted vaccination approaches to increase vaccination numbers in particular vulnerable groups,^6^ there is limited exploration of the factors which may influence the successful implementation of these activities. This study explored GM’s approaches to the implementation of targeted COVID-19 vaccination activities to reduce vaccination inequalities. The aim of this paper was to identify factors influencing the implementation of targeted vaccination approaches and draw on these to provide actionable insights to inform future vaccination delivery.

## Methods

This study was part of a larger evaluation, which also included a rapid overview of systematic reviews of interventions to address vaccine coverage in underserved, minority or vulnerable groups,^6^ and a region-wide online survey to capture data on targeted COVID-19 vaccination activities. This paper reports the qualitative component of the evaluation, which comprised interviews with key individuals involved in vaccine delivery activity. Potential participants were identified via regional contacts and sampled purposively based on their NHS and Local Authority (LA) roles and invited to interview. Participants also identified other potential participants through snowball sampling. Interviews were semi-structured and guided by a topic schedule developed from previous vaccination delivery research^6^^,^^8^ and implementation science literature.^9^ Interviews sought to capture how targeted COVID-19 vaccination activities, including those viewed as successful and less successful, were developed and implemented and what helped and hindered activity.

Interviews were carried out via Microsoft Teams by experienced researchers between February and April 2022 and were audio-recorded (voice only).

Data collection closed once the evaluation team judged that data categories were sufficiently well developed to meet the study aims.^10^ A rapid analysis approach^11^^,^^12^ was used to analyse the audio-recorded interview data collected. Data analysis was iterative and concurrent with data collection. A template based on the domains of the Consolidated Framework for Implementation Research (CFIR),^9^ enabled salient information from interviews to be rapidly documented/summarized. The template was piloted in early interviews and refined, then completed for each interview. Summaries were transferred to a matrix to give an overview of the data. Researchers exchanged summaries and reflections on interviews to discuss initial findings and extract key insights/ideas from the data.

This study was deemed service evaluation and not requiring ethics committee approval.^13^ Participants were provided with a General Data Protection Regulation compliant participant information sheet and provided verbal informed consent. All identifying details were removed from interview transcripts.

## Results

Twenty-five interviews were conducted with 27 participants (20 NHS; 7 LA), from nine of the 10 localities of GM. We were unable to recruit a participant from the tenth locality. Table 1 presents the final sample by organization.

**Table 1.** Interview participants.

GM locality	NHS respondents	LA respondents
1	2	0
2	1	0
3	1	0
4	0	1
5	3	0
6	3	2
7	4	3
8	1	0
9	5	1
	**20**	**7**

### Targeted vaccination activity overview

Participants from nine of 10 GM localities contributed information about vaccination delivery activity in their areas. All areas planned to implement some form of targeted vaccination activity, in addition to mass vaccination sites and primary care-based activities. The size and scope of targeted activities varied between localities, with more diverse areas generally offering a wider range/larger number of bespoke clinics. Many of these activities were implemented in conjunction with community groups or community/religious leaders, to help promote clinics and address community concerns.

Table 2 provides a summary of interview participants’ reports of the targeted vaccination activities taking place in Localities 1–9 (rather than a comprehensive overview of all activities). Activities are separated into those designed to: (i) increase demand and (ii) increase access; however, the two approaches were often co-ordinated and simultaneous.

**Table 2.** Example COVID-19 targeted activities across GM localities.

	Locality 1	Locality 2	Locality 3	Locality 4	Locality 5	Locality 6	Locality 7	Locality 8	Locality 9
**Targeted groups**	Housebound Care home residents Asylum seekers Individuals with learning disabilities (LD) Ethnic minority groups	Information on targeted groups limited for this locality as participant not involved in oversight of whole vaccine programme.	Housebound Individuals with LD Ethnic minority groups	Ethnic minority groups Gypsy, Roma, and Travellers	Homeless individuals Asylum seekers Housebound Ethnic and religious minority groups Individuals with LD	Homeless individuals Migrants Ethnic minority groups	Ethnic minority groups Gypsy, Roma, and Travellers Sex workers Individuals with LD Homeless individuals Asylum seekers	Less demand/push for targeted activities due to homogenous white British affluent population.	Less demand/push for targeted activities due to homogenous white British population, but recognition that vaccine coverage was not the same for all groups. Housebound Asylum seekers Care home workers Sixth form students Homeless individuals
**Targeted vaccination activities: to increase demand**	Engagement work with communities through community/ faith leaders and ‘community champions’ Door knocking by staff and volunteers to promote mobile/community venue clinics and engage with communities.	Information on targeted activity limited for this locality as participant not involved in oversight of whole vaccine programme.	Engagement with local Muslim communities. Local GPs/faith leaders encouraged those in their communities to have the vaccine.	Emphasis on engaging local communities, e.g. working with local mosque council to develop engagement strategy and working with ‘community champions’. Led to increased door knocking activity to understand why coverage was lower in some areas. Targeted activity used to complement primary care sites and to tackle misinformation.	Low uptake in Orthodox Jewish community targeted through collaboration with a Jewish ambulance group, which provided vaccinations (under the GP Federation’s governance) and carried out engagement work with the community	Additional pop-up clinics run with GPs from ethnic minority group backgrounds to enable them to have a presence and answer questions from the public. Locality decision-makers worked alongside community groups to target particular communities. Collaborated with the Jewish ambulance group to target the Orthodox Jewish community.	Emphasis on community engagement via VCFSE sector and ‘community champions’. Set-up community groups to co-design interventions to increase vaccine coverage.	Some outreach for socially deprived communities and ethnic minority groups carried out via community pharmacy. Door knocking by community groups used to encourage more vaccine coverage	Additional work carried out by door knocking to encourage vaccinations.
**Targeted vaccination activities: to increase access**	General model based on removing access barriers to disadvantaged groups. Weekly targeted clinics in areas of low coverage identified through street-level data; mainly economically deprived areas. Delivered in community venues and mobile clinics (e.g., vaccination bus).	Clinically vulnerable invited to attend early on and transport arranged for those who required it.	Primary care sites with side rooms used as ‘quiet clinics’ for individuals with LD. Also LD clinics with one vaccinator only, to reduce crowding.	Targeted clinics set-up in mosques, community centres and churches. Mobile clinics deployed in more economically deprived areas.	GP Federation responsible for targeted clinics, in collaboration with other organisations, e.g. religious community groups (clinics set-up in mosques, churches); LD specialist teams to create ‘calm’ clinics. Mobile clinics also held at university events to target sizeable student population. Most targeted clinics held in small community venues or GP practices.	Homeless individuals given access to accommodation during pandemic and offered vaccinations in these venues. Specific pop-ups created for migrants. Additional efforts to increase coverage through pop-ups in mosques, churches, markets. Not all pop-ups carried out due to cost concerns, with the primary-care sites cross-subsidising targeted clinics.	Targeted clinics set-up at community venues (mosques, churches, care homes, shopping, and community centres) and mobile clinics (a van) were utilised. To function effectively, targeted clinics required additional resources e.g. volunteers and interpreters. Some targeted elements built into clinics such as ‘calm clinics’, longer appointments, and women only clinics, which could not be offered at larger sites.	Smaller pop-up clinics set up in local community centres close to deprived areas.	Housebound individuals vaccinated by health visitors. Care home workers targeted in similar way after concerns of low coverage in this group. Clinics set-up in sixth form colleges to improve coverage in 16–18-year-olds. Homeless individuals given access to accommodation during pandemic and offered vaccinations in these venues. Specific clinics for asylum seekers and refugees enabled rapport to be built for future health work. Most targeted clinics were small, with 1–3 vaccinators. Enabled staff to have longer conversations with individuals to help them feel more comfortable.

### Factors influencing the implementation of targeted vaccination approaches

**Figure 1.**
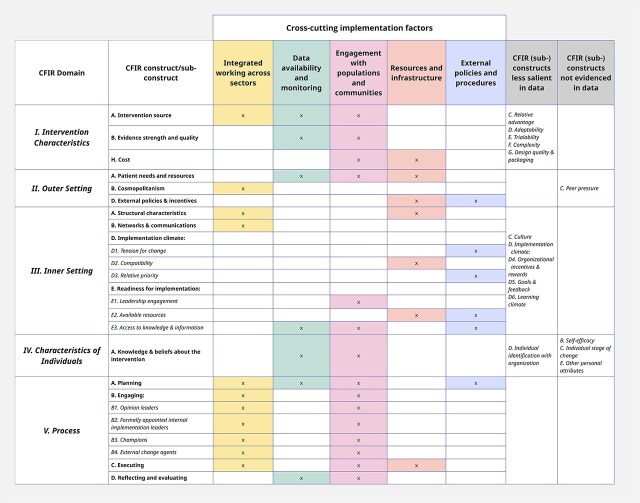
Mapping of CFIR constructs to implementation factors for targeted vaccination activities

Five factors influencing the implementation of targeted vaccination approaches were identified through analysis, informed by the CFIR, as shown in Fig. 1. These were: (i) integrated working, (ii) data availability and monitoring, (iii) engagement with populations and communities (iv) resources and infrastructure, and (v) external policies and procedures.

### Integrated working

Participants reported that they perceived local collaborative working between the health and social care system to be key to the successful delivery of the vaccination programme. Localities with established integrated structures were able to capitalize on these, such as neighbourhood place-based models. The urgency of the vaccine programme expedited the growth of existing relationships between organizations and created new ones. Across all localities, there were examples of new relationships forming between: primary care providers; primary and secondary care; and NHS and LA teams. Building partnerships with voluntary, community, faith, and social enterprise (VCFSE) sector organizations was felt to be hugely beneficial for delivery of targeted activities and sustaining these was considered important for future public health programmes.

Participants were positive about the more integrated ways of working that had been prompted by the pandemic and vaccination programme and felt that these opportunities could be capitalized on for the future:

*If you think about organizational development, I think we’ve done almost four years of organizational development in those first 12 months, in terms of people being willing to work in that way.* (R16, Locality 3, NHS).

*That goodwill and relationships and unblocking barriers, why can’t we use that going forward for more of the other challenges we have?* (R19, Locality 7, LA).

Despite this enhanced ‘spirit of collaboration’, challenges were highlighted. Competing priorities were identified, between PCNs (who led the vaccination programme) and those in public health roles (usually located in LAs), with the former prioritizing vaccination footfall and the latter prioritizing vaccine equity:

*The key for [PCNs] is to get numbers through, [but] to focus on equity rather than just numbers, you need to put a lot more resource in and so there were quite challenging conversations… We were working to try to facilitate [the equity agenda] as best we could, but at times we had very few teeth.* (R11, Locality 6, LA).

### Data availability and monitoring

The availability of good quality data was said to be a major factor in enabling targeted engagement work and vaccination activity. However, interviewees reported that data needed to be sufficiently sensitive and at the right level (e.g. data on nationality, language and religion in addition to ethnicity data), in order to detect and monitor changing patterns in vaccination coverage.

Crucially, participants emphasized that without a corresponding understanding of context, numerical data alone offered only a partial picture of community beliefs and behaviour in relation to vaccine coverage. Thus, it was said to be important to piece together insights supported by both quantitative and qualitative data to be able to map understanding of need to targeted vaccination delivery designs:

*You have to start with the data to say where the problems are and then go and speak to people to figure out what the problems are and what you should do about it …You have to iterate between the qualitative engagement and back to the data as well.* (R10, Locality 6, LA).

### Engagement with populations and communities

A thorough understanding of population characteristics/needs was seen as a crucial facilitator in targeting vaccination activity. Some localities enlisted the support of VCFSE organizations, networks, and trusted community members to act as ‘community champions’, investing in and equipping them with resources, information, training and communication tools to enable them to reach out to their own communities, to listen to questions/concerns about the vaccine and be prepared to answer these:

*…through the community champion side of the work…[that] was very much how you reach people through those more informal networks...to spread the word and we saw a real increase in uptake of the vaccine at that point.* (R25, Locality 4, LA).

In other areas with more marginalized residents, it was felt important to empower those with the knowledge and links into their populations to co-design and implement services:

*You have to give power and resources for delivering these things to people who know communities best …That’s been the big lesson for me.* (R23, Locality 7, NHS).

The siting of mobile or ‘pop-up’ clinics was said to be enhanced when planned alongside adequate engagement/communication and said to be at risk of failing without appropriate communications. Door knocking was highlighted as important by several localities, both for raising awareness of these local clinics/‘pop-up’ sites and for engaging with people from lower coverage areas and listening to their concerns; again, it was important that engagement activities involved appropriate methods and personnel.

Although less common, there were some examples of involving trusted groups, networks, and individuals in not only engagement work but also vaccine delivery. Some localities involved GPs from particularly ethic minority groups to deliver vaccinations with the aim of encouraging vaccine coverage in these groups. One locality collaborated with a Jewish ambulance service to deliver vaccinations to the Orthodox Jewish community. Delivering this service under the ambulance service’s banner rather than the NHS was said to have promoted confidence in the vaccination amongst this community.

While community engagement and feedback were seen as essential for shaping vaccination offers for diverse populations, it was described as resource-intensive and costly. Working to balance inequalities in coverage was said to generally require more resource for less return, echoing the need for ‘proportionate universalism’ in services.^7^^,^^14^

### Resources and infrastructure

The availability of resources and existing local infrastructure were key factors influencing vaccination activity approaches adopted by localities. This meant that site placement was sometimes dictated by availability and space rather than targeted to areas of local need:

*…the sites themselves were kind of selected on the basis that they were available and they were ones we thought might just about work, so there wasn’t much science in it I’m afraid, it was on a needs must basis.* (R4, Locality 6, NHS).

As sites and clinics had to be set-up from scratch, existing IT infrastructure was not available. There were reports of: equipment not working or arriving late; IT systems crashing; and limited interoperability between systems.

Workforce capacity was a huge challenge for delivery of the vaccination programme and most localities faced difficulties due to pre-pandemic workforce shortages. Flexibility in the system was considered important and localities found a range of solutions, such as sharing/re-deploying staff, bringing in volunteers and retired staff, using part-time staff to cover extra sessions, and training new vaccinators in pre-existing training hubs. Community pharmacy teams were considered particularly crucial in easing workforce pressure. Overall mobilization of the workforce to deliver the programme was largely reliant on the good-will of staff and volunteers. Long-term workforce/system planning was considered crucial to safe-guard any future delivery:

*I can’t reiterate enough how important it is to have a good workforce… if we’d [only] been in the position where we didn’t have to drag 74-year-old GPs back to vaccinate—well—that’s due to years and years of lack of investment in the workforce.* (R3, Locality 9, NHS).

### External policies and procedures

NHS England set policy and directives for the vaccination programme centrally, but the speed with which localities needed to adopt these sometimes meant that decisions were based on convenience rather than evidence. Participants reported that the planning and delivery of targeted vaccination clinics was impeded by rapid government announcements through media briefings with no prior warning or underpinning guidance/plans to meet the additional demand this triggered:

*Finding out something about the vaccination campaign from a 6 pm BBC news briefing…was really difficult to manage…. [it has] huge implications for staff on the ground…NHSE sometimes weren’t even aware of them. (*R14, Locality 6, NHS).

There was also concern that the central focus on vaccine volume and rapidity (e.g. the winter booster programme) made addressing vaccine inequity through targeted approaches more difficult for localities, with resources needing to be directed elsewhere. Payment structures for the vaccination programme were said to privilege vaccination roll-out at scale and pace, with equitable vaccine coverage as a secondary issue.

Government messaging was reported to have influenced public perceptions on the need to be vaccinated. A lack of consistent central messaging was identified as problematic by participants and was felt to have contributed to lower vaccine coverage amongst certain groups. When guidance and messaging changed, participants reported difficulties persuading groups of the validity of these changes, as these initial messages had become entrenched in the public perception and were difficult to alter.

*…the biggest issue is the constantly changing national message. So pregnant women… were told there was no real risk to them from COVID and weren’t given the COVID vaccination because we weren’t sure it was safe, trying to backtrack now on that, is horrendous.* (R9, Locality 9, NHS).

### Generation of key insights

**Figure 2.**
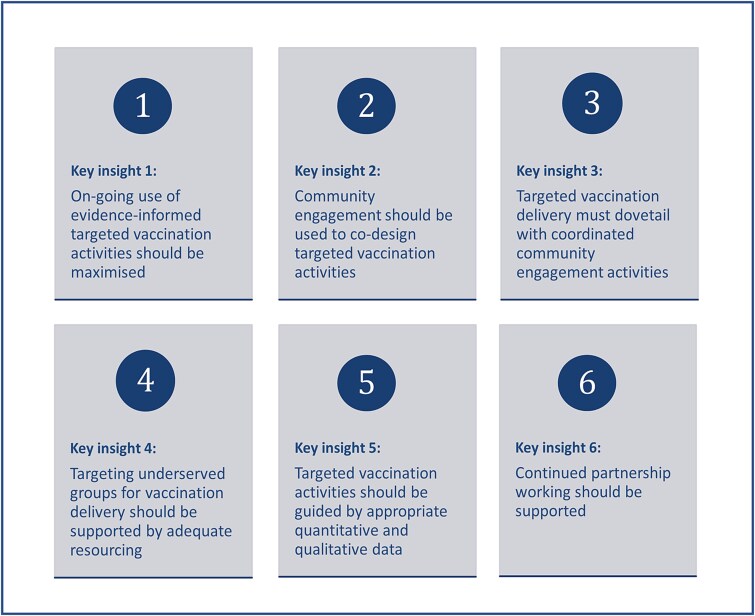
Key insights for the implementation of targeted vaccination programmes

Building on these implementation factors, we generated six key insights which provide a series of overarching principles to inform the implementation of future targeted vaccination programmes (Fig. 2). These highlight the importance of: (i) using evidence informed approaches, (ii) co-designing activities through community engagement, (iii) dovetailing vaccine delivery with community engagement, (iv) adequate resourcing for targeted approaches, (v) guiding activities with both qualitative and quantitative data, and (vi) continued partnership working.

## Discussion

### Main finding of this study

This study has identified a series of implementation factors that were influential in the delivery of targeted vaccination activity during the COVID-19 pandemic. Wider lessons for future public health interventions more generally around the need for collaborative working, adequately resourced community engagement, access to appropriate quantitative and qualitative data, long-term workforce/system planning and coherence in policy and messaging, are also highlighted. These findings have led to the generation of six key insights which form over-arching principles for the implementation of future targeted vaccination programmes.

### What is already known on this topic

Studies of the delivery of non-COVID-19 vaccinations indicate that the provision of trusted advice, community support and increased access are facilitators to vaccine uptake^15–18^ and that certain targeted vaccination activities can be effective in increasing coverage in vulnerable groups.^6^ A small number of UK and international studies that have evaluated interventions to increase COVID-19 vaccination numbers, also highlight the importance and value of targeted messaging and community engagement.^19–21^ However, there is limited research on the factors which may aid or hinder implementation of targeted activities at local level. One UK study of early COVID-19 vaccination models identified cost and resource intensiveness of targeted activities and/or community involvement as a barrier to implementation and concluded that such approaches were deprioritized in light of national performance targets.^5^^,^^22^ Our study, conducted a year later, captures a shift from the initial highly centralized national approach to the implementation of more localized forms of delivery. Our findings concur, however, that individuals involved in vaccine activity had concerns over the cost of targeted approaches and the effect of centralized messaging and directives on their ability to address vaccine inequity.

### What this study adds

This study provides in-depth insight into the experiences of NHS and LA professionals during implementation of targeted COVID-19 vaccination activities to address vaccine inequity. It reveals the importance of: building and maintaining integrated partnership working; using both quantitative and qualitative data to identify local population needs and concerns; developing culturally appropriate engagement with communities, drawing on the knowledge and connections of trusted individuals/groups; and ensuring appropriate resources and infrastructure, including workforce. It also demonstrates how a centralized directive approach can impact on localities’ ability to deliver tailored solutions.

This study is the first to identify the factors that influence the implementation of targeted COVID-19 vaccination activities, using an established implementation science framework (CFIR) to inform and guide analysis. As well as identification of these factors, this study provides a series of overarching key insights to inform future targeted vaccination activities, with general applicability to public health programmes more widely.

### Limitations of this study

Representation from some localities was low, especially from those in Local Authority roles, which resulted in perspectives from both NHS and Local Authority (which may differ) not being captured for some areas. The study coincided with the announcement of the Spring 2022 COVID-19 vaccination booster campaign, which resulted in some individuals not being available to participate due to time constraints. The study was also based on the experiences of only one region of England.

## Conclusion

In December 2023, the NHS launched a new vaccination delivery strategy for England, the principles of which align closely with these findings, citing the importance of tailored offerings, local outreach and joined-up working.^23^ The findings of this study demonstrate that successful implementation of such strategies is influenced by several factors. The factors identified, and the key insights they generated, can help to inform the design and implementation of future targeted vaccination programmes.

## Supplementary Material

Suppl_material_fdaf006

## Data Availability

The data generated and/or analysed during this study are in the form of anonymized interview notes/templates. These are not publicly available but are held on a University of Manchester secure server in line with research governance requirements. Templates are available from the corresponding author on reasonable request.

## Notes

**Fay Bradley,** Research Fellow

**Pauline A. Nelson,** Research Fellow

**Jo Dumville,** Professor of Applied Health Research
