# Supplementary material for: Implementing targeted vaccination activities to address inequalities in vaccination: a qualitative study
Source: J Public Health (Oxf). 2025 Jan 27;47(2):258–67. doi: 10.1093/pubmed/fdaf006 (PMC12123307; doi:10.1093/pubmed/fdaf006)
Supplement: Suppl_material_fdaf006 [file suppl_material_fdaf006.docx]

Supplementary material: Interview topic guide

1. **Opening question**

Please tell me about your current role – has this changed over the pandemic?

1. **Characteristics of vaccination activity**

Please tell me about the vaccine activities that you have in your locality (mass, primary care-based and bespoke?)

Can you tell me a bit more about the more ‘bespoke’ vaccination activities that you have targeted to specific groups or populations?

- What do these involve?
- Where delivered, to whom, by whom?
- When did these activities start? Frequency of operation?
- Did these activities integrate with mass vaccination/primary care delivery or were they standalone?
- What were the aims of these activities *(maybe select one or two to discuss if many?)*
  - targeting particular community groups, vaccine hesitant, increase access?
- How was that particular model designed/decided upon/chosen?
  - Who involved in design?
  - Adapted from another model elsewhere?
  - Tested/piloted elsewhere or in this locality? Is it possible to pilot?
  - Has this model been evaluated or evidenced?
  - Why do you think this model is suited to the locality – why should it work?
  - Why was this model considered better than others available?
- Did this require lots of additional resource (e.g. additional staff, buildings, kit etc.)?

1. **Characteristics of the outer setting**

- Why is there a need for local bespoke vaccine activities?
  - What do you think are the main barriers to vaccination uptake in the local community? *Access, hesitancy?*
- How will/do these activities meet local need?
- Have you had any user-feedback from those accessing these vaccine activities?
- Are other localities adopting same/similar models?
- How does this affect support for implementing the model in your locality?
- Are there any local needs that are not being met by these activities currently?
  - Gaps in provision – hard to reach groups?
  - Are there any plans to address this in the future?

1. **Characteristics of the inner setting**

- Do you think there is anything about the locality in terms of existing infrastructure and networks that affects implementation of these activities? Facilitates or hinders?
- Are the activities compatible with the existing system and infrastructure? Integration?
- Do these activities replace existing provision, complement or conflict with it?
- Are there/will there be any infrastructure changes needed to implement these activities?
- How essential are local bespoke vaccine activities? Where do they sit on the priority list currently?
- Have you received endorsement for these activities from community leaders? Who are they? What types of endorsement? Has it had any affect?
- What resource is needed to deliver these activities? Have you been able to secure these? – how straight forward/challenging?
- Are there any training needs in relation to their delivery? How addressed?

1. **Characteristics of individuals (focusing on targeted populations)**

- What are the local area demographics like?
- In your opinion, what are the community’s thoughts towards C-19 vaccination?
- *Have different members of your community held different views about the vaccine?*
- *What are the main reasons for taking up the vaccine, what are the main concerns towards the vaccine?*
- *Has there been any shift in views about the vaccine?*
- How do these views compare with views on other vaccines, e.g. flu?

1. **Process**

- Would you say the vaccination model/activity went to plan? If not what changed?
- Are there any particular elements that enhanced success (e.g. engaging with key stakeholders/champions)?
- Are there any particular elements that contributed to the plan not working?
- Are you monitoring implementation and activity? If so how?
- What are the key lessons learnt from this experience to share with others?

1. **Closing question**

Is there anything else that is important for us to know about vaccination activity in your locality that we have not already discussed?
